# Methyl 3-(4-isopropyl­phen­yl)-1-phenyl-3,3a,4,9b-tetra­hydro-1*H*-chromeno[4,3-*c*]isoxazole-3a-carboxyl­ate

**DOI:** 10.1107/S1600536811026365

**Published:** 2011-07-09

**Authors:** J. Kanchanadevi, G. Anbalagan, J. Srinivasan, M. Bakthadoss, V. Manivannan

**Affiliations:** a Department of Physics, Velammal Institute of Technology, Panchetty, Chennai 601 204, India; b Department of Physics, Presidency College (Autonomous), Chennai 600 005, India; c Department of Organic Chemistry, University of Madras, Maraimalai Campus, Chennai 600 025, India; d Department of Research and Development, PRIST University, Vallam, Thanjavur 613 403, Tamil Nadu, India

## Abstract

In the title compound, C_27_H_27_NO_4_, the five-membered isoxazole ring adopts an envelope conformation and the six-membered pyran ring adopts a half-chair conformation. The dihedral angle between the mean planes of the isoxazole ring and the chromene ring system is 54.95 (4)°.

## Related literature

For the biological activity of chromenopyrrole, see: Caine (1993 ▶) and of benzopyran and isoxazolidine, see: Lin *et al.* (1996 ▶); Hu *et al.* (2004 ▶). For related structures, see: Gangadharan *et al.* (2011 ▶); Swaminathan *et al.* (2011 ▶).
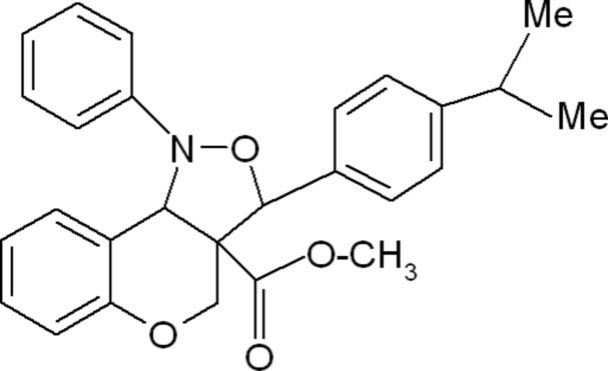


## Experimental

### 

#### Crystal data

C_27_H_27_NO_4_
*M* *_r_* = 429.50
Triclinic, 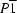

*a* = 9.3555 (3) Å
*b* = 10.7247 (4) Å
*c* = 12.0449 (4) Å
α = 94.707 (1)°
β = 104.730 (1)°
γ = 96.385 (1)°
*V* = 1153.88 (7) Å^3^
*Z* = 2
Mo *K*α radiation
μ = 0.08 mm^−1^
*T* = 295 K
0.35 × 0.30 × 0.25 mm

#### Data collection

Bruker Kappa APEXII CCD diffractometer
Absorption correction: multi-scan (*SADABS*; Sheldrick, 1996 ▶) *T*_min_ = 0.937, *T*_max_ = 0.954
32129 measured reflections
8565 independent reflections
4738 reflections with *I*=2σ(*I*)
*R*_int_ = 0.030

#### Refinement

*R*[*F* ^2^ > 2σ(*F* ^2^)] = 0.054
*wR*(*F* ^2^) = 0.166
*S* = 1.03
8565 reflections
292 parameters
H-atom parameters constrained
Δρ_max_ = 0.21 e Å^−3^
Δρ_min_ = −0.23 e Å^−3^

### 

Data collection: *APEX2* (Bruker, 2004 ▶); cell refinement: *SAINT* (Bruker, 2004 ▶); data reduction: *SAINT*; program(s) used to solve structure: *SHELXS97* (Sheldrick, 2008 ▶); program(s) used to refine structure: *SHELXL97* (Sheldrick, 2008 ▶); molecular graphics: *PLATON* (Spek, 2009 ▶); software used to prepare material for publication: *SHELXL97*.

## Supplementary Material

Crystal structure: contains datablock(s) global. DOI: 10.1107/S1600536811026365/pv2423sup1.cif

Additional supplementary materials:  crystallographic information; 3D view; checkCIF report

## Figures and Tables

**Fig. 1.**
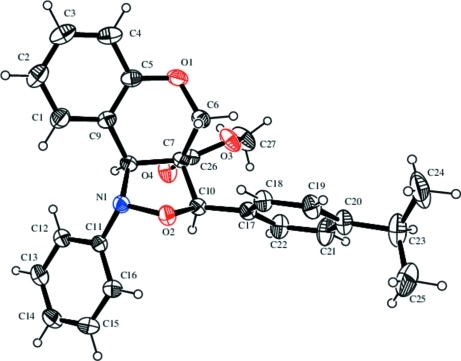
The molecular structure of the title compound with atom labels and 30% probability displacement ellipsoids for non-H atoms.

## supplementary crystallographic information

### Comment

Chromenopyrrole compounds are used in the treatment of impulsive disorders (Caine, 1993). It is well known that benzopyran and isoxazolidine derivatives possess interesting biological and pharmacological activities (Lin *et al.*, 1996; Hu *et al.*, 2004).

The geometric parameters of the title molecule (Fig. 1) agree well with the corresponding geometric parameters reported in closely related structures (Gangadharan *et al.*, 2011; Swaminathan *et al.*, 2011). The dihedral angle between the two benzene rings [(C11—C16) and (C17—C22)] is 73.02 (2) °. The sum of bond angles around N1 [335.04 (9) °]indicates the *sp^3^* hybridization state of atom N1 in the molecule. The molecular structure is stabilized by weak intramolecular C—H···O interactions.

### Experimental

A mixture of (*E*)-methyl 2-((2-formylphenoxy)methyl)-3-(4-isopropylphenyl) acrylate (2 mmol, 0.68 g) and *N*-phenylhydroxylamine (3 mmol, 0.33 g) in ethanol (10 ml) was refluxed for 6 h. After the completion of the reaction as indicated by TLC, the reaction mixture was concentrated and the resulting crude mass was diluted with water (15 ml) and extracted with ethyl acetate (3x15 ml). The combined organic layer was washed with brine (3x15 ml) and dried over anhydrous Na_2_SO_4_, solvent was removed under reduced pressure. The crude mass was purified by column chromatography on silica gel (Acme 100–200 mesh), using ethyl acetate-hexane (0.5: 9.5) to afford the title compound as a colourless solid in 84% yield. The compound was recrystallised from ethyl acetate to produce X-ray diffraction quality crystals.

### Refinement

H atoms were positioned geometrically and refined using riding model with C—H distances = 0.93, 0.96, 0.97 and 0.98 Å for aryl, methyl, methylene and methine type H-atoms, respectively, using *U*_iso_(H) = 1.2*U*_eq_(non-methyl C atoms) and 1.5*U*_eq_(methyl C atoms).

### Crystal data

**Table:**

C_27_H_27_NO_4_	*Z* = 2
*M**_r_* = 429.50	*F*(000) = 456
Triclinic, *P*1	*D*_x_ = 1.236 Mg m^−^^3^
Hall symbol: -P 1	Mo *K*α radiation, λ = 0.71073 Å
*a* = 9.3555 (3) Å	Cell parameters from 8942 reflections
*b* = 10.7247 (4) Å	θ = 2.3–27.5°
*c* = 12.0449 (4) Å	µ = 0.08 mm^−^^1^
α = 94.707 (1)°	*T* = 295 K
β = 104.730 (1)°	Block, colourless
γ = 96.385 (1)°	0.35 × 0.30 × 0.25 mm
*V* = 1153.88 (7) Å^3^	

### Data collection

**Table:**

Bruker Kappa APEXII CCD diffractometer	8565 independent reflections
Radiation source: fine-focus sealed tube	4738 reflections with *I*=2σ(*I*)
graphite	*R*_int_ = 0.030
Detector resolution: 0 pixels mm^-1^	θ_max_ = 33.0°, θ_min_ = 2.3°
ω and φ scans	*h* = −14→14
Absorption correction: multi-scan (*SADABS*; Sheldrick, 1996)	*k* = −16→16
*T*_min_ = 0.937, *T*_max_ = 0.954	*l* = −18→17
32129 measured reflections	

### Refinement

**Table:**

Refinement on *F*^2^	Primary atom site location: structure-invariant direct methods
Least-squares matrix: full	Secondary atom site location: difference Fourier map
*R*[*F*^2^ > 2σ(*F*^2^)] = 0.054	Hydrogen site location: inferred from neighbouring sites
*wR*(*F*^2^) = 0.166	H-atom parameters constrained
*S* = 1.03	*w* = 1/[σ^2^(*F*_o_^2^) + (0.0735*P*)^2^ + 0.1032*P*] where *P* = (*F*_o_^2^ + 2*F*_c_^2^)/3
8565 reflections	(Δ/σ)_max_ = 0.001
292 parameters	Δρ_max_ = 0.21 e Å^−^^3^
0 restraints	Δρ_min_ = −0.23 e Å^−^^3^

### Fractional atomic coordinates and isotropic or equivalent isotropic displacement parameters (Å^2^)

**Table:**

	*x*	*y*	*z*	*U*_iso_*/*U*_eq_	
C1	0.60704 (18)	0.16741 (14)	0.53375 (12)	0.0569 (3)	
H1	0.5311	0.2173	0.5296	0.068*	
C2	0.6510 (2)	0.13476 (16)	0.43502 (13)	0.0680 (4)	
H2	0.6063	0.1639	0.3656	0.082*	
C3	0.7615 (2)	0.05886 (15)	0.44040 (14)	0.0676 (4)	
H3	0.7895	0.0350	0.3738	0.081*	
C4	0.83058 (18)	0.01821 (14)	0.54304 (14)	0.0614 (4)	
H4	0.9048	−0.0333	0.5461	0.074*	
C5	0.78884 (15)	0.05468 (12)	0.64292 (12)	0.0487 (3)	
C6	0.87307 (13)	0.10646 (12)	0.84153 (11)	0.0460 (3)	
H6A	0.9191	0.1884	0.8303	0.055*	
H6B	0.9345	0.0801	0.9110	0.055*	
C7	0.71742 (12)	0.11725 (10)	0.85621 (10)	0.0375 (2)	
C8	0.61543 (13)	0.15211 (10)	0.74339 (10)	0.0389 (2)	
H8	0.5169	0.1021	0.7291	0.047*	
C9	0.67386 (14)	0.12726 (11)	0.63853 (10)	0.0439 (3)	
C10	0.71737 (13)	0.23270 (10)	0.94285 (10)	0.0395 (2)	
H10	0.6250	0.2215	0.9674	0.047*	
C11	0.45688 (14)	0.31910 (11)	0.77135 (10)	0.0430 (3)	
C12	0.32924 (15)	0.25929 (14)	0.69002 (12)	0.0537 (3)	
H12	0.3366	0.1947	0.6362	0.064*	
C13	0.19070 (17)	0.29527 (17)	0.68844 (14)	0.0670 (4)	
H13	0.1060	0.2542	0.6338	0.080*	
C14	0.1775 (2)	0.39055 (19)	0.76652 (15)	0.0755 (5)	
H14	0.0845	0.4138	0.7659	0.091*	
C15	0.3036 (2)	0.45114 (18)	0.84575 (15)	0.0786 (5)	
H15	0.2955	0.5166	0.8985	0.094*	
C16	0.44312 (18)	0.41693 (14)	0.84892 (13)	0.0611 (4)	
H16	0.5274	0.4596	0.9030	0.073*	
C17	0.84729 (13)	0.26602 (11)	1.04857 (11)	0.0421 (3)	
C18	0.97498 (14)	0.34429 (12)	1.04776 (12)	0.0487 (3)	
H18	0.9834	0.3755	0.9796	0.058*	
C19	1.09029 (15)	0.37650 (13)	1.14762 (13)	0.0552 (3)	
H19	1.1746	0.4302	1.1455	0.066*	
C20	1.08295 (16)	0.33070 (13)	1.25029 (13)	0.0587 (4)	
C21	0.95492 (18)	0.25349 (16)	1.25008 (14)	0.0690 (4)	
H21	0.9470	0.2217	1.3181	0.083*	
C22	0.83775 (16)	0.22189 (14)	1.15152 (12)	0.0588 (4)	
H22	0.7521	0.1707	1.1545	0.071*	
C23	1.2070 (2)	0.36685 (18)	1.36204 (16)	0.0839 (6)	
H23	1.1953	0.3028	1.4138	0.101*	
C24	1.3610 (2)	0.3669 (3)	1.3421 (2)	0.1332 (10)	
H24A	1.3655	0.2878	1.3005	0.200*	
H24B	1.4349	0.3785	1.4151	0.200*	
H24C	1.3798	0.4345	1.2979	0.200*	
C25	1.1916 (3)	0.4915 (3)	1.4210 (2)	0.1299 (10)	
H25A	1.2074	0.5571	1.3740	0.195*	
H25B	1.2643	0.5086	1.4946	0.195*	
H25C	1.0933	0.4887	1.4321	0.195*	
C26	0.64937 (13)	−0.00295 (11)	0.89318 (10)	0.0404 (3)	
C27	0.6998 (2)	−0.16605 (17)	1.01174 (18)	0.0825 (6)	
H27A	0.6501	−0.1412	1.0689	0.124*	
H27B	0.7834	−0.2070	1.0468	0.124*	
H27C	0.6315	−0.2233	0.9510	0.124*	
N1	0.59985 (11)	0.28810 (9)	0.76661 (9)	0.0423 (2)	
O1	0.86540 (10)	0.01709 (9)	0.74473 (9)	0.0559 (2)	
O2	0.71185 (9)	0.33448 (8)	0.87192 (8)	0.0470 (2)	
O3	0.75166 (11)	−0.05530 (10)	0.96424 (10)	0.0651 (3)	
O4	0.51902 (11)	−0.04183 (10)	0.86632 (9)	0.0648 (3)	

### Atomic displacement parameters (Å^2^)

**Table:**

	*U*^11^	*U*^22^	*U*^33^	*U*^12^	*U*^13^	*U*^23^
C1	0.0691 (9)	0.0559 (8)	0.0467 (7)	0.0098 (7)	0.0167 (6)	0.0054 (6)
C2	0.0911 (12)	0.0681 (10)	0.0451 (7)	0.0029 (9)	0.0221 (7)	0.0055 (7)
C3	0.0884 (11)	0.0617 (9)	0.0595 (9)	−0.0048 (8)	0.0418 (8)	−0.0040 (7)
C4	0.0672 (9)	0.0558 (8)	0.0708 (10)	0.0050 (7)	0.0401 (8)	−0.0014 (7)
C5	0.0512 (7)	0.0446 (7)	0.0552 (7)	0.0037 (5)	0.0250 (6)	0.0035 (5)
C6	0.0371 (6)	0.0510 (7)	0.0523 (7)	0.0081 (5)	0.0145 (5)	0.0092 (5)
C7	0.0348 (5)	0.0371 (5)	0.0421 (6)	0.0062 (4)	0.0121 (4)	0.0057 (4)
C8	0.0386 (5)	0.0354 (5)	0.0438 (6)	0.0066 (4)	0.0123 (5)	0.0049 (4)
C9	0.0498 (7)	0.0398 (6)	0.0435 (6)	0.0036 (5)	0.0169 (5)	0.0025 (5)
C10	0.0362 (5)	0.0372 (6)	0.0443 (6)	0.0033 (4)	0.0099 (5)	0.0053 (5)
C11	0.0502 (7)	0.0420 (6)	0.0387 (6)	0.0168 (5)	0.0095 (5)	0.0088 (5)
C12	0.0521 (7)	0.0584 (8)	0.0479 (7)	0.0177 (6)	0.0057 (6)	0.0019 (6)
C13	0.0533 (8)	0.0830 (11)	0.0625 (9)	0.0245 (8)	0.0038 (7)	0.0109 (8)
C14	0.0684 (10)	0.0972 (13)	0.0708 (10)	0.0476 (10)	0.0190 (8)	0.0172 (9)
C15	0.0887 (12)	0.0819 (11)	0.0700 (10)	0.0486 (10)	0.0180 (9)	−0.0039 (9)
C16	0.0691 (9)	0.0578 (8)	0.0535 (8)	0.0260 (7)	0.0070 (7)	−0.0044 (6)
C17	0.0391 (6)	0.0373 (6)	0.0470 (6)	0.0030 (5)	0.0071 (5)	0.0037 (5)
C18	0.0443 (6)	0.0479 (7)	0.0510 (7)	−0.0009 (5)	0.0094 (5)	0.0095 (5)
C19	0.0431 (7)	0.0484 (7)	0.0655 (9)	−0.0052 (5)	0.0028 (6)	0.0107 (6)
C20	0.0564 (8)	0.0504 (8)	0.0573 (8)	−0.0019 (6)	−0.0054 (6)	0.0135 (6)
C21	0.0689 (10)	0.0738 (10)	0.0526 (8)	−0.0130 (8)	−0.0008 (7)	0.0245 (7)
C22	0.0522 (7)	0.0617 (8)	0.0551 (8)	−0.0108 (6)	0.0064 (6)	0.0146 (6)
C23	0.0793 (12)	0.0758 (11)	0.0705 (11)	−0.0177 (9)	−0.0212 (9)	0.0270 (9)
C24	0.0680 (13)	0.171 (3)	0.129 (2)	0.0246 (15)	−0.0369 (13)	0.0206 (19)
C25	0.1180 (19)	0.143 (2)	0.0866 (15)	−0.0023 (17)	−0.0268 (14)	−0.0302 (15)
C26	0.0416 (6)	0.0378 (6)	0.0444 (6)	0.0072 (5)	0.0154 (5)	0.0044 (5)
C27	0.0738 (11)	0.0735 (11)	0.1208 (16)	0.0249 (9)	0.0416 (11)	0.0604 (11)
N1	0.0438 (5)	0.0388 (5)	0.0418 (5)	0.0082 (4)	0.0063 (4)	0.0032 (4)
O1	0.0552 (5)	0.0592 (6)	0.0633 (6)	0.0236 (4)	0.0262 (5)	0.0091 (5)
O2	0.0469 (5)	0.0359 (4)	0.0518 (5)	0.0033 (3)	0.0024 (4)	0.0053 (4)
O3	0.0478 (5)	0.0641 (6)	0.0934 (8)	0.0159 (4)	0.0223 (5)	0.0444 (6)
O4	0.0472 (5)	0.0686 (6)	0.0718 (7)	−0.0101 (5)	0.0062 (5)	0.0244 (5)

### Geometric parameters (Å, °)

**Table:**

C1—C9	1.3846 (19)	C14—H14	0.9300
C1—C2	1.385 (2)	C15—C16	1.386 (2)
C1—H1	0.9300	C15—H15	0.9300
C2—C3	1.377 (2)	C16—H16	0.9300
C2—H2	0.9300	C17—C22	1.3831 (19)
C3—C4	1.371 (2)	C17—C18	1.3841 (17)
C3—H3	0.9300	C18—C19	1.3853 (18)
C4—C5	1.3959 (18)	C18—H18	0.9300
C4—H4	0.9300	C19—C20	1.382 (2)
C5—O1	1.3703 (17)	C19—H19	0.9300
C5—C9	1.3893 (18)	C20—C21	1.378 (2)
C6—O1	1.4289 (16)	C20—C23	1.526 (2)
C6—C7	1.5265 (16)	C21—C22	1.3840 (19)
C6—H6A	0.9700	C21—H21	0.9300
C6—H6B	0.9700	C22—H22	0.9300
C7—C26	1.5235 (16)	C23—C25	1.501 (3)
C7—C8	1.5492 (16)	C23—C24	1.520 (3)
C7—C10	1.5525 (16)	C23—H23	0.9800
C8—N1	1.4925 (15)	C24—H24A	0.9600
C8—C9	1.5144 (16)	C24—H24B	0.9600
C8—H8	0.9800	C24—H24C	0.9600
C10—O2	1.4378 (14)	C25—H25A	0.9600
C10—C17	1.5060 (16)	C25—H25B	0.9600
C10—H10	0.9800	C25—H25C	0.9600
C11—C16	1.3858 (18)	C26—O4	1.1957 (14)
C11—C12	1.3887 (18)	C26—O3	1.3211 (15)
C11—N1	1.4274 (16)	C27—O3	1.4484 (17)
C12—C13	1.389 (2)	C27—H27A	0.9600
C12—H12	0.9300	C27—H27B	0.9600
C13—C14	1.369 (2)	C27—H27C	0.9600
C13—H13	0.9300	N1—O2	1.4350 (13)
C14—C15	1.371 (3)		
			
C9—C1—C2	121.34 (14)	C16—C15—H15	119.3
C9—C1—H1	119.3	C11—C16—C15	119.93 (15)
C2—C1—H1	119.3	C11—C16—H16	120.0
C3—C2—C1	119.49 (15)	C15—C16—H16	120.0
C3—C2—H2	120.3	C22—C17—C18	118.30 (12)
C1—C2—H2	120.3	C22—C17—C10	119.56 (11)
C4—C3—C2	120.59 (14)	C18—C17—C10	122.09 (11)
C4—C3—H3	119.7	C17—C18—C19	120.57 (12)
C2—C3—H3	119.7	C17—C18—H18	119.7
C3—C4—C5	119.60 (14)	C19—C18—H18	119.7
C3—C4—H4	120.2	C20—C19—C18	121.50 (13)
C5—C4—H4	120.2	C20—C19—H19	119.3
O1—C5—C9	121.49 (11)	C18—C19—H19	119.3
O1—C5—C4	117.81 (12)	C21—C20—C19	117.34 (13)
C9—C5—C4	120.70 (13)	C21—C20—C23	120.08 (14)
O1—C6—C7	110.68 (10)	C19—C20—C23	122.53 (14)
O1—C6—H6A	109.5	C20—C21—C22	121.90 (14)
C7—C6—H6A	109.5	C20—C21—H21	119.0
O1—C6—H6B	109.5	C22—C21—H21	119.0
C7—C6—H6B	109.5	C17—C22—C21	120.37 (13)
H6A—C6—H6B	108.1	C17—C22—H22	119.8
C26—C7—C6	111.81 (9)	C21—C22—H22	119.8
C26—C7—C8	111.35 (9)	C25—C23—C24	111.4 (2)
C6—C7—C8	110.09 (9)	C25—C23—C20	110.72 (16)
C26—C7—C10	110.01 (9)	C24—C23—C20	112.07 (18)
C6—C7—C10	111.99 (9)	C25—C23—H23	107.5
C8—C7—C10	101.13 (8)	C24—C23—H23	107.5
N1—C8—C9	111.90 (9)	C20—C23—H23	107.5
N1—C8—C7	105.99 (9)	C23—C24—H24A	109.5
C9—C8—C7	113.50 (9)	C23—C24—H24B	109.5
N1—C8—H8	108.4	H24A—C24—H24B	109.5
C9—C8—H8	108.4	C23—C24—H24C	109.5
C7—C8—H8	108.4	H24A—C24—H24C	109.5
C1—C9—C5	118.20 (12)	H24B—C24—H24C	109.5
C1—C9—C8	121.15 (11)	C23—C25—H25A	109.5
C5—C9—C8	120.46 (11)	C23—C25—H25B	109.5
O2—C10—C17	109.26 (9)	H25A—C25—H25B	109.5
O2—C10—C7	102.26 (9)	C23—C25—H25C	109.5
C17—C10—C7	118.65 (9)	H25A—C25—H25C	109.5
O2—C10—H10	108.7	H25B—C25—H25C	109.5
C17—C10—H10	108.7	O4—C26—O3	123.99 (11)
C7—C10—H10	108.7	O4—C26—C7	124.51 (11)
C16—C11—C12	118.53 (12)	O3—C26—C7	111.39 (10)
C16—C11—N1	121.18 (12)	O3—C27—H27A	109.5
C12—C11—N1	119.98 (11)	O3—C27—H27B	109.5
C11—C12—C13	120.50 (13)	H27A—C27—H27B	109.5
C11—C12—H12	119.7	O3—C27—H27C	109.5
C13—C12—H12	119.7	H27A—C27—H27C	109.5
C14—C13—C12	120.70 (15)	H27B—C27—H27C	109.5
C14—C13—H13	119.7	C11—N1—O2	111.46 (9)
C12—C13—H13	119.7	C11—N1—C8	118.02 (10)
C13—C14—C15	118.93 (15)	O2—N1—C8	105.56 (8)
C13—C14—H14	120.5	C5—O1—C6	111.86 (9)
C15—C14—H14	120.5	N1—O2—C10	105.57 (8)
C14—C15—C16	121.40 (15)	C26—O3—C27	116.86 (11)
C14—C15—H15	119.3		
			
C9—C1—C2—C3	1.2 (2)	O2—C10—C17—C18	−29.12 (15)
C1—C2—C3—C4	−1.7 (2)	C7—C10—C17—C18	87.45 (14)
C2—C3—C4—C5	−0.3 (2)	C22—C17—C18—C19	0.4 (2)
C3—C4—C5—O1	−176.94 (13)	C10—C17—C18—C19	177.75 (12)
C3—C4—C5—C9	2.7 (2)	C17—C18—C19—C20	0.9 (2)
O1—C6—C7—C26	68.26 (13)	C18—C19—C20—C21	−1.2 (2)
O1—C6—C7—C8	−56.07 (13)	C18—C19—C20—C23	−178.72 (15)
O1—C6—C7—C10	−167.75 (9)	C19—C20—C21—C22	0.3 (3)
C26—C7—C8—N1	130.21 (10)	C23—C20—C21—C22	177.81 (16)
C6—C7—C8—N1	−105.20 (10)	C18—C17—C22—C21	−1.4 (2)
C10—C7—C8—N1	13.38 (11)	C10—C17—C22—C21	−178.79 (14)
C26—C7—C8—C9	−106.59 (11)	C20—C21—C22—C17	1.1 (3)
C6—C7—C8—C9	18.01 (13)	C21—C20—C23—C25	−94.8 (2)
C10—C7—C8—C9	136.58 (10)	C19—C20—C23—C25	82.6 (3)
C2—C1—C9—C5	1.1 (2)	C21—C20—C23—C24	140.1 (2)
C2—C1—C9—C8	−173.87 (13)	C19—C20—C23—C24	−42.5 (2)
O1—C5—C9—C1	176.55 (12)	C6—C7—C26—O4	−147.55 (13)
C4—C5—C9—C1	−3.1 (2)	C8—C7—C26—O4	−23.93 (16)
O1—C5—C9—C8	−8.42 (19)	C10—C7—C26—O4	87.35 (15)
C4—C5—C9—C8	171.93 (11)	C6—C7—C26—O3	36.03 (14)
N1—C8—C9—C1	−51.91 (15)	C8—C7—C26—O3	159.65 (10)
C7—C8—C9—C1	−171.81 (11)	C10—C7—C26—O3	−89.07 (12)
N1—C8—C9—C5	133.21 (12)	C16—C11—N1—O2	20.92 (16)
C7—C8—C9—C5	13.31 (16)	C12—C11—N1—O2	−165.45 (11)
C26—C7—C10—O2	−152.76 (9)	C16—C11—N1—C8	143.32 (13)
C6—C7—C10—O2	82.24 (11)	C12—C11—N1—C8	−43.05 (16)
C8—C7—C10—O2	−34.95 (10)	C9—C8—N1—C11	123.40 (11)
C26—C7—C10—C17	87.01 (12)	C7—C8—N1—C11	−112.39 (11)
C6—C7—C10—C17	−37.99 (14)	C9—C8—N1—O2	−111.26 (10)
C8—C7—C10—C17	−155.18 (10)	C7—C8—N1—O2	12.96 (11)
C16—C11—C12—C13	−1.4 (2)	C9—C5—O1—C6	−30.67 (16)
N1—C11—C12—C13	−175.17 (13)	C4—C5—O1—C6	148.99 (12)
C11—C12—C13—C14	0.3 (2)	C7—C6—O1—C5	63.65 (13)
C12—C13—C14—C15	0.7 (3)	C11—N1—O2—C10	92.31 (10)
C13—C14—C15—C16	−0.7 (3)	C8—N1—O2—C10	−37.00 (11)
C12—C11—C16—C15	1.4 (2)	C17—C10—O2—N1	171.97 (9)
N1—C11—C16—C15	175.13 (14)	C7—C10—O2—N1	45.40 (10)
C14—C15—C16—C11	−0.4 (3)	O4—C26—O3—C27	−0.3 (2)
O2—C10—C17—C22	148.15 (12)	C7—C26—O3—C27	176.14 (13)
C7—C10—C17—C22	−95.27 (15)		

### Hydrogen-bond geometry (Å, °)

**Table:**

*D*—H···*A*	*D*—H	H···*A*	*D*···*A*	*D*—H···*A*
C6—H6B···O3	0.97	2.37	2.706 (2)	100
C8—H8···O4	0.98	2.35	2.846 (2)	111
C16—H16···O2	0.93	2.38	2.713 (2)	101
